# The association between misperceptions around weight status and quality of life in adults in Australia

**DOI:** 10.1186/s12955-017-0627-7

**Published:** 2017-03-21

**Authors:** Christopher Heard, Paul A. Scuffham, Julie Ratcliffe, Jennifer A. Whitty

**Affiliations:** 1 School of Pharmacy, Faculty of Health and Behavioural Sciences, The University of Queensland, St Lucia, QLD Australia; 2 Menzies Health Institute Queensland, Griffith University, Queensland, Australia; 3 Institute for Choice, Business School, University of South Australia, Adelaide, South Australia Australia; 4 Health Economics Group, Norwich Medical School, Faculty of Medicine and Health Sciences, University of East Anglia, Norwich Research Park, Norwich, UK NR4 7JT

**Keywords:** Obesity, Perceived weight, Predictors of quality-of-life, Public health

## Abstract

**Background:**

Limited evidence supports a possible association between a person’s perception of their weight status and their quality of life (QoL). This study evaluates whether *mis*perception around weight status is associated with QoL and the impact of gender on this association.

**Methods:**

A cross-sectional survey of Australian adults (*n* = 1,905 analysed) collected self-reported height and weight (used to estimate BMI), gender and QoL (described using the AQoL-8D). Participants reported whether they perceived their weight status to be ‘underweight’, ‘healthy weight’, ‘overweight’ or ‘obese’. Misperception around weight status was categorised based on perceived weight status and self-reported BMI. Ordinary least squares regression was used to test associations between self-reported overall, physical and psychosocial QoL, misperception of weight status, and gender, across different BMI categories, after controlling for income, education, relationship status and health conditions.

**Results:**

Compared to accurate perception, underestimation of weight status was associated with higher overall QoL for obese males and females and for overweight males. Overestimation of weight status was associated with higher overall QoL for underweight females and lower overall QoL for healthy weight males and females. The same pattern was seen for psychosocial QoL. Physical QoL was less sensitive to misperception than psychosocial QoL.

**Conclusions:**

Self-reported misperception around weight status is associated with overall, psychosocial and to a lesser extent physical QoL in Australian adults, although its role depends on BMI category and gender. Generally misperception in the direction of “healthy weight” is associated with higher QoL and overestimation of weight status by those who are of healthy weight is associated with lower QoL. Findings should be confirmed in datasets that contain measured as opposed to self-report height and weight.

**Electronic supplementary material:**

The online version of this article (doi:10.1186/s12955-017-0627-7) contains supplementary material, which is available to authorized users.

## Background

The worldwide prevalence of obesity has more than doubled since 1980. Australia now has the fifth highest obesity rate among Organisation for Economic Co-operation and Development countries [1]. The health burden of overweight and obesity in Australia is reflected in the economic burden, with overweight and obesity estimated to cost over $10 billion Australian dollars (AU$) annually [2]. Patient-reported outcomes including generic health-related quality of life (QoL) measures are increasingly used to evaluate both population health and health outcomes. Given the increasing health and economic burden associated with obesity, developing an understanding of the predictors of QoL is important in studying obesity and its management as a public health issue.

There is a negative association between overweight/obesity (usually measured by Body Mass Index, BMI) and QoL in adults [3–6], adolescents [7–9] and older adults [10]. Understanding this association is complicated because many people misperceive their weight status [11–15], with some authors arguing that there has been a ‘normalisation’ of higher weight which encourages misperception [11]. Perception of weight status may also have health consequences, which may exceed the negative associations between actual BMI and QoL. Large cross-sectional surveys of Australian adults (*n* = 17,253), Dutch adults (*n* = 4,501) and adolescents in Mexico (*n* = 2,401) have reported perceived weight status to be associated with either psychological distress [16] or QoL [17, 18], independent of the effect of BMI.

Recent studies have also begun to explore the associations between *mis*perception of weight and health status. Overweight American adults who misperceive their weight are less likely to pursue weight-loss, and often engage in less physical activity [14]. Studies examining the association between misperceptions and health status in Canadian adults (*n* = 87,545) [15], children in Iran (*n* = 5,570) [19] and Australian adolescents (*n* = 3,040) [20] suggest that people of healthy weight who misperceive their weight in either direction (i.e. to be under or overweight) report poorer health [15, 19] and that misperceptions may be associated with better health in some people who perceive themselves to be of “about right” weight status, even if their BMI is outside the healthy weight range [15, 20]. However, research has been limited in this area. For example, few studies have measured the impact of (mis)perception on a validated measure of QoL which can be considered to be a patient-reported health outcome [17, 18, 20]. Of those that have done so only one study undertaken in the Netherlands has been in adults [17]; and, this study explored the effects of weight perception but not misperception on QoL. Previous studies assessing the association between (mis)perceived weight and QoL have undertaken little exploration of whether this association differs according to different dimensions of QoL. Yet, the reported links between weight, body image and psychosocial factors suggest we might expect the impact of (mis)perceived weight status to be stronger for the mental than physical dimensions of QoL [21–23].

Moreover, the potential role of gender in explaining the association between weight (mis)perception and health status has been raised. Studies suggest weight perception and related behaviour differ by gender. Specifically, overweight and obese men are less likely to have accurate weight perception or attempt weight loss than women [15, 24]. However, studies exploring whether any relationship between weight misperception and QoL differs by gender are inconclusive. Whilst an Australian study found a lack of association between weight misperception (described as perception that was “incorrect with BMI”, direction of misperception not specified) and psychological distress which was uniform for men and women [16], a Dutch study found the association between the perception of being overweight and reduced QoL to be particularly strong for women [17]. However, this study did not explore the effect of weight *mis*perception and therefore the impact of gender on this association. The only study we are aware of that has explored whether the association between weight misperception and QoL varied by gender was undertaken in Australian adolescents [20]. It found that the association between BMI and QoL is moderated according to weight perception; specifically, adolescents who were outside the healthy weight range and misperceived their weight as being “about right” reported a higher HRQoL than adolescents whose weight perception was concordant with their actual weight status. The relationship differed by gender; however, the exact nature of the differences by gender and the QoL domains that differ were sensitive to the analytic method (regression analysis or average marginal effects analysis). That is, the associations with gender differed across the two analysis approaches employed.

The current study builds on this relatively small literature by examining the association of misperception of weight status with the QoL of Australian adults, and whether this association varies by QoL dimension (psychosocial versus physical) or gender.

## Methods

### Survey

This study reports a secondary analysis of data from a large, cross-sectional online survey undertaken as part of a parent study which aimed to explore the public’s preferences around priority setting in health care including in the context of bariatric surgery [25–27]. The survey provided some background information on obesity and collected a range of sociodemographic, health status and perceived weight measures which are described in Measures.

### Participants

A total of 1,994 adults (≥18 years) from Queensland and South Australia were recruited from an online survey panel (Pureprofile®). Quotas ensured representativeness of the adult population by age and gender for each State. All adults included in the online survey panel residing in these two states were invited to participate by email and received a small incentive for participation. There were no exclusion criteria (until a specific age/gender quota was filled, at which point adults of this age/gender were no longer eligible). However, 89 participants were excluded due to missing data (4.5% of the original sample), leaving 1,905 participants included in the analysis. Excluded participants were more likely to report annual household incomes below AU$40,000 and less likely to have visited a GP or hospital between one and three times in the preceding 12 months than included participants.

### Measures

The survey collected a range of measures, which are described below.

*QoL score –* The Assessment of Quality of Life (AQoL-8D) questionnaire, which consists of 35 items across 8 domains with up to 6 responses per item, was used to measure QoL [28]. Responses were combined into an overall preference-based measure of QoL using the Australian utility weight tariff, called a utility score, ranging between 0 (indicating a state equivalent to being dead) and 1 (indicating perfect health) [29]. The physical super dimension consists of three dimensions (pain, sensory perception, and independence in mobility and self-care) and the psychosocial super dimension consists of five dimensions (mental health, relationships, coping, self-worth and happiness) [28]. The super dimensions were scored on a scale that is not preference-based from “Dimension worst health state” (scored 0) to “Dimension best health state” (scored 1).

*Self-reported height and weight –* This was used to estimate self-reported Body Mass Index (BMI). Participants were categorised as ‘underweight’ (BMI < 18.5 kg/m^2^), ‘healthy weight’ (BMI 18.5 to <25 kg/m^2^), ‘overweight’ (BMI 25 to <30 kg/m^2^) or ‘obese’ (BMI ≥ 30 kg/m^2^) based on World Health Organisation (WHO) standards [31].

*Weight perception –* Participants responded to the question “Do you perceive yourself as being ‘underweight’, ‘healthy weight’, ‘overweight’ or ‘obese’?” This measure is consistent with the approach taken in previous studies measuring weight perception (e.g. [14–17]. These categories were chosen to match the BMI categories and weight classifications used in WHO standards [31].

*Covariates –* Participants provided information about a range of characteristics that described the sample in relation to the Australian population, and which could be expected to be associated with their QoL, BMI and/or perceived weight. These were their recent health history (visits to hospitals and general practitioners), weight-related comorbidities (diabetes, heart disease, hypertension, osteoarthritis), gender, marital status, highest level of completed education and annual gross household income.

### Statistical analysis

Stata Statistical Software 13 (StataCorp, College Station, Texas, USA) was used for all statistical analysis with Microsoft Excel 2010 (Microsoft, Redmond, Washington, USA) used for the production of figures. Kappa statistics were used to assess the agreement between weight perceptions and self-reported BMI categories [15, 32]. Prior to the regression analysis the data were weighted so that gender and age proportions matched those reported in the 2011 Australian Census [33]. Weight misperception was categorised based on self-reported weight perception and self-reported BMI, according to whether the participant’s perceived weight category was ‘correct’, an ‘overestimation’ or an ‘underestimation’.

### Primary analysis

Multiple linear regression was used to estimate the effects of weight misperception and its interaction with gender and self-reported BMI category, on overall QoL. The model controlled for the effects of age and comorbidities as these are known to be important for explaining QoL scores [34, 35]. Additionally, indicator variables for income group, education and marital status were included to control for the importance of socioeconomic factors [12, 13, 36]. Controlling for these variables is also consistent with previous work in this area by Herman et al. [15]. Self-reported hospital admissions and General Practitioner (GP) visits were also included as variables to control for ‘general health’ not explained by the comorbidities. This gave the following model for estimation, where the covariates and their codes are as defined in Table 1:

**Table 1:** Participant Characteristics

Characteristic	Variable code	Males (*n* = 921)		Females (*n* = 984)		Total (*n* = 1 905)	
		Mean (SD)		Mean (SD)		Mean (SD)	
Age (years)	Age (continuous variable)	46.8 (16.5)		46.3 (16.2)		46.6 (16.4)	
BMI (kg/m^2^)	Not applicable (entered as weight categories)	28.3 (6.7)		27.9 (8.0)		28.1 (7.4)	
QoL (AQoL-8D)							
- Utility score	Dependent variable	0.70 (0.21)		0.68 (0.21)		0.69 (0.21)	
- Psychosocial score	Dependent variable	0.40 (0.21)		0.37 (0.19)		0.38 (0.20)	
- Physical score	Dependent variable	0.70 (0.21)		0.69 (0.22)		0.70 (0.22)	
		N	%	n	%	n	%
Marital status							
- Never married	Referent	218	24	184	19	402	21
- Divorced	divorced	75	8	126	13	201	11
- Widowed	widowed	12	1	47	5	59	3
- Married	married	616	67	627	64	1 243	65
Education (completed Year 12)	gradhs	708	77	709	72	1 417	74
Household income (per annum; Australian dollars)							
- < $40,000	Referent	216	23	263	27	479	25
- $40,001–$70,000	Inc40k70k	236	26	230	23	466	24
- $70,001–$100,000	Inc70k100k	164	18	181	18	345	18
- $100,001–$130,000	Inc100k130k	109	12	77	8	186	10
- > $130,000	Incg130k	86	9	93	9	179	9
- other	incother	110	12	140	14	250	13
Hospital admissions							
- 0 admissions	Referent	724	79	793	81	1 517	80
- 1–3 admissions	Hospadmit3	189	21	187	19	376	20
- >3 admissions	Hospadmit4	8	1	4	0	12	1
GP visits							
- 0 visits	Referent	149	16	85	9	234	12
- 1–3 visits	Gp3	502	55	516	52	1 018	53
- >3 visits	Gp4	270	29	383	39	653	34
Weight perception							
- Underweight	Not applicable (entered as weight misperception)	35	4	33	3	68	4
- Healthy	Not applicable (entered as weight misperception)	386	42	433	44	819	43
- Overweight	Not applicable (entered as weight misperception)	373	40	365	37	738	39
- Obese	Not applicable (entered as weight misperception)	127	14	153	16	280	15
Weight category (from self-reported BMI in kg/m^2^)							
- Underweight (<18.5)	trueuw	45	5	100	10	145	8
- Healthy weight (18.5 to <25)	Referent	270	29	340	35	610	32
- Overweight (25 to <30)	trueow	320	35	245	25	565	30
-Obese (≥30)	trueob	286	31	299	30	585	31
Weight misperception							
- Underestimate	underestweight	296	32	224	23	520	27
- Overestimate	overestweight	50	5	133	14	183	10
Comorbidities							
- Diabetes	diabetes	109	12	93	9	202	11
- Heart disease	heart	90	10	53	5	143	8
- Hypertension	highbp	238	26	224	23	462	24
- Arthritis	arthritis	98	11	179	18	277	15

*BMI* Body Mass Index, *GP* General Practitioner, *QoL* quality of life, *SD* standard deviation

$$ \begin{array}{l}\widehat{QoL}={\beta}_0+{\beta}_1 age+{\beta}_2 female+{\beta}_3 inc40 k70 k+{\beta}_4 inc70 k100 k+{\beta}_5 inc100 k130 k\\ {}\begin{array}{cc}\hfill \hfill & \hfill \begin{array}{l}\kern6em +{\beta}_6 incg130 k+{\beta}_7 incother+{\beta}_8 hospadmit3+{\beta}_9 hospadmit4+{\beta}_{10} gp3\\ {}\kern6em +{\beta}_{11} gp4+{\beta}_{12} diabetes+{\beta}_{13} heart+{\beta}_{14} highbp+{\beta}_{15} arthritis\\ {}\kern6em +{\beta}_{16} gradhs+{\beta}_{17} married+{\beta}_{18} divorced+{\beta}_{19} widowed+{\gamma}_1 trueuw\\ {}\kern6em +{\gamma}_2 trueow+{\gamma}_3 trueob+{\delta}_1 underestweight+{\delta}_2 overestweight\\ {}\kern6em +{\eta}_1 trueuw\times female+{\eta}_2 trueow\times female+{\eta}_3 trueob\times female\\ {}\kern6em +{\lambda}_1 trueuw\times overestweight+{\lambda}_2 trueow\times underestweight\\ {}\kern6em +{\lambda}_3 trueow\times overestweight+{\lambda}_4 trueob\times underestweight\\ {}\kern6em +{\mu}_1 trueuw\times overestweight\times female+{\mu}_2 trueow\times underestweight\\ {}\kern6em \times female+{\mu}_3 trueow\times overestweight\times female+{\mu}_4 trueob\\ {}\kern6em \times underestweight\times female\end{array}\hfill \end{array}\end{array} $$

The coefficients of interest were those of misperception indicator variables, gender and self-reported BMI class, and the two- and three-way interactions between these variables. Two-way interactions between misperception and BMI class allowed assessment of whether misperceptions in or against the direction of healthy weight are associated with QoL. Interactions (up to three-way) with gender allowed us to examine whether any of the effects are different between males and females. The relevant coefficients were added together to obtain estimates of the combined effects of misperception and self-reported BMI class by gender. For example, the estimated combined effect of misperception and BMI for an overweight woman who underestimates her weight, relative to a woman of healthy weight who correctly perceives her weight is:

$$ {\gamma}_2+{\delta}_1+{\eta}_2+{\lambda}_2+{\mu}_2 $$

Forest plots were constructed to visualise these effects.

### Secondary analysis

Two additional models were estimated as described above, in which the physical and psychosocial super dimension scores respectively replaced the overall QoL scores as the dependent variables.

## Results

### Demographic and health characteristics

Participants had a mean age of 46.6 years (SD 16.4), and 984 (51.7%) were female (Table 1). Mean QoL was 0.69 (SD 0.21) for the overall utility score, 0.38 (SD 0.20) for the psychosocial dimension and 0.70 (SD 0.22) for the physical dimension, and was similar for males and females. Mean self-reported BMI was in the overweight range for both genders. One third (32%) of males underestimated their weight status compared to 23% of females, while 5% of males overestimated their weight status compared to 14% of females.

Agreement between perceived weight status and self-reported BMI category (Table 2) was moderate (kappa 0.47) and comparable for females (kappa 0.49) and males (kappa 0.46). Only 31% (45 of 145) of underweight participants correctly assessed their weight status. Participants of healthy weight typically assessed their weight correctly (524 of 610; 86%), but 31% (175 of 565) overweight participants assessed themselves to be of a healthy weight and 51% (297 of 585) obese participants incorrectly assessed themselves as being overweight. Overall, 36% (295 of 819) participants who assessed themselves as being of a healthy weight were not. Most (95% of underweight, 100% of healthy weight, 99% of overweight and 92% of obese) participants who misperceived their weight did so by only one BMI category.

**Table 2:** Perceived weight status according to self-reported BMI

Perceived weight status	Number of participants (%) reporting perceived weight category, by Self-reported BMI Category (kg/m^2)^				
	Underweight (<18.5)	Healthy weight (18.5 to <25)	Overweight (25 to <30)	Obese (≥30)	Total
Underweight	45 (31.0%)	20 (3.3%)	2 (0.4%)	1 (0.2%)	68 (3.6%)
Healthy weight	95 (65.5%)	524 (85.9%)	175 (31.0%)	25 (4.3%)	819 (43.0%)
Overweight	4 (2.8%)	66 (10.8%)	371 (65.7%)	297 (50.8%)	738 (38.7%)
Obese	1 (0.7%)	0 (0.0%)	17 (3.0%)	262 (44.8%)	280 (14.7%)
Total	145 (100.0%)	610 (100.0%)	565 (100.0%)	585 (100.0%)	1905 (100.0%)

### The association between misperception of weight status and QoL

The regression results are reported as supplementary information. Most of the sociodemographic variables were significantly associated with overall, psychosocial and physical QoL (*p* < 0.05), the exception being gender. *Arthritis* was the only comorbidity significantly associated with overall or physical QoL; no comorbidities were associated with psychosocial QoL.

Based on the individual regression results (Additional file 1), being overweight or obese was significantly associated with lower overall, psychosocial and physical QoL, compared to being healthy weight. Being underweight, was associated with lower psychosocial QoL, but was not observed to be associated with physical or overall QoL. Overestimating BMI was significantly associated with lower overall, psychosocial and physical QoL. This was not the case for underestimating BMI. However, the effect of weight misperception on QoL was moderated by self-reported weight status. Overall, overestimating BMI was associated with higher overall QoL in those who were underweight and lower overall QoL in those who were healthy weight or overweight. Underestimating BMI was associated with higher overall QoL in those who were overweight or obese, but was not observed to be associated with overall QoL in those who were healthy weight. A similar pattern was observed for psychosocial and physical QoL, but not all observations reached significance at the 5% level. When considered as an individual variable in the model, gender was not observed to moderate the association of misperception on overall, psychosocial or physical QoL. However, we retain the gender interaction terms due to the importance of gender in previous studies and the fact that our interest is in total effects on QoL, not individual coefficients.

**Fig. 1:**
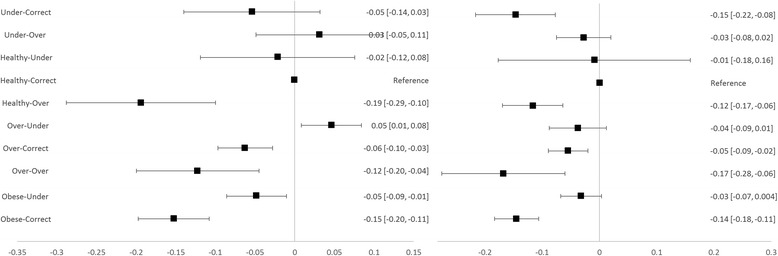
Overall QoL Effects for Males (*left*) and Females (*right*) with 95% CIs (labels are self-reported BMI category-perceived weight class)

**Fig. 2:**
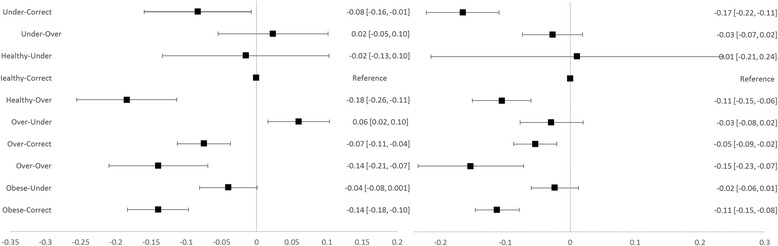
Psychosocial QoL Effects for Males (*left*) and Females (*right*) with 95% CIs (labels are self-reported BMI category-perceived weight class)

**Fig. 3:**
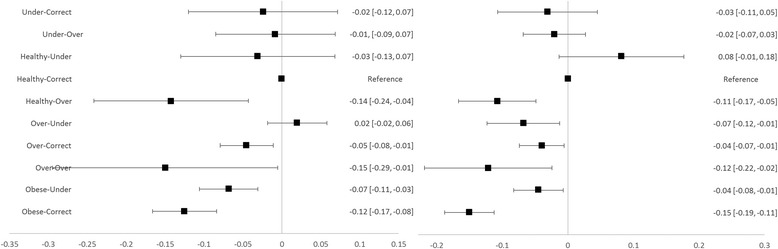
Physical QoL Effects for Males (*left*) and Females (*right*) with 95% CIs (labels are self-reported BMI category-perceived weight class)

Average total QoL effects of weight-perception combinations are presented by gender with 95% confidence intervals in forest plots at Figs. 1, 2 and 3. Compared to accurate perception, underestimation of BMI was associated with higher overall QoL for obese males and females and for overweight males (Fig. 1, difference in mean utility score of 0.10 to 0.11). Overestimation of BMI was associated with higher overall QoL for underweight females (difference in mean utility score of 0.12) and lower overall QoL for healthy weight males and females (difference in mean utility score of 0.12 to 0.19).

The same pattern was seen for psychosocial QoL (Fig. 2) as for overall QoL. Compared to accurate perception, underestimation of BMI was associated with higher psychosocial QoL for obese males and females and for overweight males (difference in mean psychosocial score of 0.09 to 0.13). Overestimation of BMI was associated with higher psychosocial QoL for underweight females (difference in mean psychosocial score of 0.14) and lower psychosocial QoL for healthy weight males and females (difference in mean psychosocial score of 0.11 to 0.18).

Physical QoL (Fig. 3) was less sensitive to misperception than overall or psychosocial QoL. Specifically compared to accurate perception, underestimation of BMI was only observed to be associated with higher physical QoL for obese males (difference in mean physical score of 0.11). Overestimation of BMI was not observed to be associated with higher physical QoL for any group, but was observed to be associated with lower physical QoL for healthy weight males and females (difference in mean physical score of 0.11 to 0.14).

## Discussion

Overall, we find evidence to support the argument that self-perception is an important moderator of the relationship between self-reported BMI and QoL in Australian adults, but that this association varies by BMI category and to a lesser extent, gender. We also find that the importance of misperception differs between overweight and obese adults. Previous studies have combined overweight and obese into one weight category [15, 19, 20]. While this offers advantages (the overweight/obese distinction may be unclear to participants) our findings suggest that the severity of overweight may be important. Our study suggests rates of accurate weight perception among Australian males and females of healthy weight are high (87 and 85% respectively) and are similar to rates reported in Canadian (82% M, 77% F) [15] and USA (about 75% M, 65% F) [11–13] studies. Nonetheless, misperceptions about weight appears to be common particularly among people outside the healthy weight range, with 36% (24%) of overweight males (females) assessing themselves to be of a healthy weight and only 44% (25%) of underweight males (females) assessing their weight correctly in our study. Misperception is therefore likely to be an important consideration for understanding the effects of underweight, overweight and obesity in Australia.

While underestimation does not appear to be associated with lower QoL in adults of healthy weight, we find evidence that overestimation of weight is associated with lower psychosocial, physical and overall QoL for adults of healthy weight, for both males and females. Previous studies in a range of age groups and national and cultural backgrounds have generally found that misperception is associated with lower QoL for people of healthy weight and may moderate the relationship between weight and QoL for people outside the healthy weight range [15, 17–20]. A study of Canadian adults found that people of healthy weight who correctly perceived their weight were least likely to report suboptimal QoL indicators and that misperception of weight in any direction was associated with higher likelihood of suboptimal QoL indicators [15]. However, other studies suggest the direction of misperception may be important. In an Australian study [20]; underweight, overweight and obese adolescents who perceived themselves as being of a healthy weight reported higher QoL than adolescents who correctly perceived themselves to be outside the healthy weight range.

In our study, misperception of weight was associated with a utility score for some categories that was 0.10 to 0.19 lower than a person of the same self-reported BMI category who correctly perceived their weight. This exceeds the 0.04 to 0.075 reduction in utility score that is reported to reflect an important change in health status [30], and is similar to the change in utility score observed to be associated with an increase of greater than 20 years in age, or a reduction in household income to less than AU$40,000, in the current study. This implies that the health impacts associated with misperception of weight status are substantial at a population level, particularly when considered alongside the high prevalence of misperceived weight status. A detriment in utility score of 0.10 to 0.19 equates to a loss of one quality-adjusted life year (QALY, defined as a year of life lived in perfect health), for every 5 to 10 years lived with that misperception. Thus, understanding the causes of misperception, its association with health outcomes, and addressing weight misperception through public health, weight management and psychological interventions, has the potential to make a very substantial impact on both individual and population health.

Whilst misperception was associated with lower QoL for some groups in our study, misperception in the ‘direction’ of healthy weight was associated with higher QoL for some groups. This phenomenon has been reported previously [20]. However, our results extend this finding to adults and also suggest a more important role for gender than previous studies. Hayward et al. [20] found that adolescents’ perceptions of underweight were associated with reduced physical functioning scores in males but not in females. We do not find underestimation of weight category by adults of healthy weight to be associated with lower QoL for either gender. Instead, we find an important role for gender in moderating the influence of perception in overweight people who are not obese. Whilst underestimating weight is associated with a higher QoL in obese individuals of either gender, we find evidence that overweight males who underestimate their weight status also report a higher overall and psychosocial QoL, while overweight females do not appear to do so. The reasons why this gender difference is observed may be complex and related to cultural and social norms regarding body image, and would be worthy of further research. However, given the self-reported data, it is possible that in some cases these overweight males are not actually overweight (if body composition were measured) and if so they may in fact have accurate perceptions; whilst overweight females may be less likely to have this mis-categorization of weight status by BMI. Conversely, we find that overestimation of weight status is associated with higher overall and psychosocial (but not physical) QoL in underweight females but not males. Overall, these findings suggest there may be an important role for gender in moderating the association between BMI, weight perception and QoL which has not been previously described.

This cross-sectional study is unable to assess causality. However, it is important that further research evaluate causality, as our findings suggest important policy implications. If misperceptions in the direction of healthy weight are protective against adverse QoL consequences for those who are not in the healthy weight range, public health programmes must consider the potential negative consequences of correcting misperceptions and ensure appropriate support is made available. This has been argued by Hayward et al. [20] in the context of adolescent programmes; our results support an extension of this argument to also apply to programmes aimed at adults - particularly obese people and overweight males. Nevertheless, any potential impact of correcting misperception of weight on QoL needs to be balanced against the health and economic benefits of encouraging people to take action to achieve a healthy weight. This challenging task has not been considered in this study. It also needs to be balanced against the potential benefits of correcting misperceptions that are associated with lower QoL in certain groups, requiring careful targeting of any interventions. Future research may tackle this, for example, by further exploring and confirming the effect of weight misperception on overall and dimensional QoL and other health outcomes, in other population groups as well as causality. Ultimately, this growing literature suggests that weight intervention studies should measure outcomes that consider the impact of the intervention on weight perception as well as on weight status.

This study utilises a substantial data set based on the general community dwelling population in Australia in which QoL was measured using a validated instrument. In so doing, it adds a more nuanced understanding to an area of great importance for public health. It also extends our understanding by describing previously unreported differences in the way that misperceptions by male and female adults are associated with QoL. However, there are potential limitations to our study. First, BMI was estimated based on self-reported weight and height. Although this is a common approach [15, 16], height and weight would ideally be independently measured [19, 20]. Nevertheless, the similarity of our findings to those from previous studies and substantial agreement recorded between self-report and measured BMI in a number of studies [41–43, 45] suggests that this shortcoming may not be severe (although small but significant underreporting of BMI is known to occur particularly in the severely obese, adolescent girls, older adults especially men, and some survey modes [41–47]). Second, there were some differences in the characteristics of our sample as compared to adults in Australia. The sample in our study was similar to Australian norms in gender, age and education [37], but on average had a lower household income [39] and lower health status [40] than adults in Australia. Nevertheless, the proportion self-reporting a BMI in the overweight or obese range was similar to rates in Australian adults; although, the proportion self-reporting a BMI in the underweight range was high (7.6%) compared to 1.5% of Australian adults [38]. As our study was based on secondary analysis, our data were missing potentially important variables. We lacked information about smoking and exercise, which affects both general health and weight, and could not be adjusted for in the model [48]. It is unclear to what extent these differences might impact generalisability, and our findings which should be confirmed in other populations.

## Conclusions

Weight management at a population level is a difficult task complicated by the complex psychological and social consequences and causes of obesity. Since improved quality of life is an objective of public health programmes (see, for example, the evolution of the Healthy People strategic framework in the USA [49]) there is value in understanding the pathways by which weight status can affect QoL. This study presents evidence of the importance of weight misperception as a determinant of QoL in Australian adults and further extends a growing literature dedicated to understanding this relationship. Findings indicate that misperception around one’s own weight status is associated with overall, physical and most especially psychosocial QoL in Australian adults, although its role depends on BMI category and gender. This may have significant policy implications for public health research and programmes that target the prevention or management of obesity.

## Additional file

Additional file 1: Table S1. Linear Regression - QoL (utility score) as the dependent variable. **Table S2.** Linear Regression – Psychosocial QoL as the dependent variable. **Table S3.** Linear Regression – Physical QoL as the dependent variable. (DOCX 31 kb)

## Abbreviations

AQoL-8D: Assessment of quality of life 8 dimension questionnaire
AU$: Australian dollars
BMI: Body mass index
CI: Confidence interval
F: Female
GP: General practitioner
M: Male
QALY: Quality-adjusted life year
QoL: Health-related quality of life
SD: Standard deviation
USA: United States of America
WHO: World Health Organisation
