# Supplementary material for: The association between misperceptions around weight status and quality of life in adults in Australia
Source: Health Qual Life Outcomes. 2017 Mar 21;15:53. doi: 10.1186/s12955-017-0627-7 (PMC5359799; doi:10.1186/s12955-017-0627-7)
Supplement: Additional file 1: Table S1. — Linear Regression - QoL (utility score) as the dependent variable. Table S2. Linear Regression – Psychosocial QoL as the dependent variable. Table S3. Linear Regression – Physical QoL as the dependent variable. (DOCX 31 kb) [file 12955_2017_627_MOESM1_ESM.docx]

**Additional file 1**

**Table S1: Linear Regression - QoL (utility score) as the dependent variable**

|  | Coefficient | | SE | t | P>\|t\| | [95% Conf. Interval] | |
| --- | --- | --- | --- | --- | --- | --- | --- |
| age | 0.002 | | 0.000 | 7.14 | 0.000 | 0.002 | 0.003 |
| female | -0.002 | | 0.016 | -0.11 | 0.910 | -0.032 | 0.029 |
| inc40k70k | 0.042 | | 0.013 | 3.28 | 0.001 | 0.017 | 0.067 |
| inc70k100k | 0.070 | | 0.014 | 5.01 | 0.000 | 0.042 | 0.097 |
| inc100k130k | 0.067 | | 0.017 | 3.95 | 0.000 | 0.034 | 0.100 |
| incg130k | 0.119 | | 0.016 | 7.22 | 0.000 | 0.087 | 0.151 |
| incother | 0.048 | | 0.015 | 3.09 | 0.002 | 0.017 | 0.078 |
| hospadmit3 | -0.049 | | 0.012 | -4.11 | 0.000 | -0.072 | -0.025 |
| hospadmit4 | -0.122 | | 0.064 | -1.90 | 0.057 | -0.248 | 0.004 |
| gp3 | 0.005 | | 0.014 | 0.39 | 0.694 | -0.022 | 0.033 |
| gp4 | -0.072 | | 0.016 | -4.45 | 0.000 | -0.104 | -0.040 |
| gradhs | 0.031 | | 0.010 | 3.02 | 0.003 | 0.011 | 0.051 |
| divorced | -0.016 | | 0.018 | -0.90 | 0.366 | -0.051 | 0.019 |
| widowed | 0.025 | | 0.029 | 0.86 | 0.388 | -0.032 | 0.081 |
| married | 0.031 | | 0.012 | 2.48 | 0.013 | 0.006 | 0.055 |
| trueuw | -0.054 | | 0.044 | -1.23 | 0.219 | -0.140 | 0.032 |
| trueow | -0.062 | | 0.018 | -3.55 | 0.000 | -0.097 | -0.028 |
| trueob | -0.153 | | 0.023 | -6.67 | 0.000 | -0.198 | -0.108 |
| diabetes | 0.003 | | 0.016 | 0.21 | 0.836 | -0.029 | 0.036 |
| heart | -0.012 | | 0.020 | -0.60 | 0.548 | -0.050 | 0.027 |
| highbp | -0.002 | | 0.012 | -0.13 | 0.893 | -0.025 | 0.022 |
| arthritis | -0.042 | | 0.014 | -3.08 | 0.002 | -0.069 | -0.015 |
| overestweight | -0.194 | | 0.048 | -4.04 | 0.000 | -0.288 | -0.100 |
| underestwei~t | -0.021 | | 0.050 | -0.43 | 0.666 | -0.119 | 0.076 |
| overestuw | 0.279 | | 0.075 | 3.72 | 0.000 | 0.132 | 0.425 |
| overestow | 0.134 | | 0.062 | 2.15 | 0.031 | 0.012 | 0.256 |
| underestow | 0.130 | | 0.054 | 2.43 | 0.015 | 0.025 | 0.235 |
| underestob | 0.126 | | 0.056 | 2.27 | 0.023 | 0.017 | 0.235 |
| gintuw | -0.093 | | 0.056 | -1.65 | 0.099 | -0.203 | 0.018 |
| gintow | 0.008 | | 0.025 | 0.31 | 0.760 | -0.041 | 0.056 |
| gintob | 0.008 | | 0.030 | 0.27 | 0.791 | -0.050 | 0.066 |
| gintoveruw | -0.043 | | 0.089 | -0.48 | 0.631 | -0.217 | 0.132 |
| gintoverow | -0.130 | | 0.087 | -1.49 | 0.136 | -0.302 | 0.041 |
| gintunderow | -0.104 | | 0.105 | -0.99 | 0.321 | -0.310 | 0.101 |
| gintunderob | -0.004 | | 0.104 | -0.04 | 0.969 | -0.209 | 0.201 |
| gintoverest | 0.077 | | 0.055 | 1.40 | 0.161 | -0.031 | 0.185 |
| gintunderest | 0.012 | | 0.099 | 0.12 | 0.901 | -0.182 | 0.207 |
| _cons | 0.578 | | 0.026 | 22.67 | 0.000 | 0.528 | 0.629 |
| Number observations=1905 | | |  |  |  |  |  |
| F(37,1868) = 15.78 | |  |  |  |  |  |  |
| Prob>F=0.0000 | |  |  |  |  |  |  |
| R-squared=0.2229 | |  |  |  |  |  |  |

**Table S2: Linear Regression – Psychosocial QoL as the dependent variable**

|  | Coefficient | SE | t | P>\|t\| | [95% Conf. Interval] | |
| --- | --- | --- | --- | --- | --- | --- |
| age | 0.003 | 0.000 | 8.99 | 0.000 | 0.002 | 0.004 |
| female | -0.021 | 0.018 | -1.18 | 0.239 | -0.056 | 0.014 |
| inc40k70k | 0.034 | 0.012 | 2.75 | 0.006 | 0.010 | 0.058 |
| inc70k100k | 0.058 | 0.014 | 4.21 | 0.000 | 0.031 | 0.084 |
| inc100k130k | 0.051 | 0.017 | 3.04 | 0.002 | 0.018 | 0.084 |
| incg130k | 0.106 | 0.017 | 6.12 | 0.000 | 0.072 | 0.141 |
| incother | 0.042 | 0.015 | 2.78 | 0.005 | 0.012 | 0.072 |
| hospadmit3 | -0.036 | 0.011 | -3.32 | 0.001 | -0.058 | -0.015 |
| hospadmit4 | -0.113 | 0.048 | -2.37 | 0.018 | -0.207 | -0.019 |
| gp3 | -0.003 | 0.015 | -0.18 | 0.857 | -0.032 | 0.027 |
| gp4 | -0.056 | 0.017 | -3.37 | 0.001 | -0.088 | -0.023 |
| gradhs | 0.025 | 0.010 | 2.51 | 0.012 | 0.006 | 0.045 |
| divorced | -0.007 | 0.017 | -0.42 | 0.673 | -0.040 | 0.026 |
| widowed | 0.005 | 0.026 | 0.18 | 0.856 | -0.046 | 0.055 |
| married | 0.027 | 0.012 | 2.26 | 0.024 | 0.004 | 0.051 |
| trueuw | -0.084 | 0.039 | -2.15 | 0.031 | -0.160 | -0.007 |
| trueow | -0.075 | 0.019 | -3.88 | 0.000 | -0.113 | -0.037 |
| trueob | -0.140 | 0.022 | -6.35 | 0.000 | -0.184 | -0.097 |
| diabetes | 0.004 | 0.016 | 0.28 | 0.778 | -0.026 | 0.035 |
| heart | -0.014 | 0.019 | -0.77 | 0.444 | -0.052 | 0.023 |
| highbp | 0.001 | 0.012 | 0.11 | 0.916 | -0.022 | 0.024 |
| arthritis | -0.010 | 0.013 | -0.74 | 0.460 | -0.035 | 0.016 |
| overestweight | -0.184 | 0.036 | -5.09 | 0.000 | -0.256 | -0.113 |
| underestweight | -0.015 | 0.060 | -0.26 | 0.798 | -0.134 | 0.103 |
| overestuw | 0.292 | 0.063 | 4.63 | 0.000 | 0.168 | 0.415 |
| overestow | 0.120 | 0.051 | 2.37 | 0.018 | 0.021 | 0.219 |
| underestow | 0.150 | 0.064 | 2.34 | 0.019 | 0.024 | 0.276 |
| underestob | 0.116 | 0.065 | 1.79 | 0.073 | -0.011 | 0.242 |
| gintuw | -0.082 | 0.048 | -1.71 | 0.088 | -0.176 | 0.012 |
| gintow | 0.021 | 0.026 | 0.81 | 0.415 | -0.029 | 0.071 |
| gintob | 0.028 | 0.028 | 1.01 | 0.312 | -0.026 | 0.082 |
| gintoveruw | -0.048 | 0.075 | -0.64 | 0.523 | -0.195 | 0.099 |
| gintoverow | -0.114 | 0.069 | -1.64 | 0.101 | -0.250 | 0.022 |
| gintunderow | -0.136 | 0.134 | -1.01 | 0.311 | -0.399 | 0.127 |
| gintunderob | -0.038 | 0.133 | -0.29 | 0.776 | -0.300 | 0.224 |
| gintoverest | 0.079 | 0.043 | 1.85 | 0.065 | -0.005 | 0.163 |
| gintunderest | 0.027 | 0.130 | 0.20 | 0.838 | -0.228 | 0.281 |
| _cons | 0.253 | 0.026 | 9.60 | 0.000 | 0.202 | 0.305 |
| Number observations=1905 | |  |  |  |  |  |
| F(37,1868) = 13.41 | |  |  |  |  |  |
| Prob>F=0.0000 |  |  |  |  |  |  |
| R-squared=0.1824 | |  |  |  |  |  |

**Table S3: Linear Regression – Physical QoL as the dependent variable**

|  | Coefficient | SE | t | P>\|t\| | [95% Conf. Interval] | |
| --- | --- | --- | --- | --- | --- | --- |
| age | -0.001 | 0.000 | -2.50 | 0.013 | -0.001 | 0.000 |
| female | 0.017 | 0.015 | 1.16 | 0.245 | -0.012 | 0.047 |
| inc40k70k | 0.033 | 0.013 | 2.59 | 0.010 | 0.008 | 0.059 |
| inc70k100k | 0.060 | 0.014 | 4.25 | 0.000 | 0.032 | 0.088 |
| inc100k130k | 0.063 | 0.017 | 3.62 | 0.000 | 0.029 | 0.097 |
| incg130k | 0.094 | 0.017 | 5.68 | 0.000 | 0.062 | 0.126 |
| incother | 0.039 | 0.015 | 2.61 | 0.009 | 0.010 | 0.069 |
| hospadmit3 | -0.052 | 0.012 | -4.17 | 0.000 | -0.076 | -0.027 |
| hospadmit4 | -0.104 | 0.072 | -1.44 | 0.150 | -0.245 | 0.038 |
| gp3 | 0.001 | 0.014 | 0.05 | 0.958 | -0.026 | 0.028 |
| gp4 | -0.088 | 0.016 | -5.38 | 0.000 | -0.121 | -0.056 |
| gradhs | 0.028 | 0.010 | 2.76 | 0.006 | 0.008 | 0.049 |
| divorced | -0.034 | 0.018 | -1.89 | 0.058 | -0.070 | 0.001 |
| widowed | 0.036 | 0.030 | 1.21 | 0.228 | -0.023 | 0.095 |
| married | 0.013 | 0.012 | 1.03 | 0.305 | -0.011 | 0.036 |
| trueuw | -0.024 | 0.049 | -0.50 | 0.621 | -0.120 | 0.072 |
| trueow | -0.045 | 0.017 | -2.61 | 0.009 | -0.079 | -0.011 |
| trueob | -0.125 | 0.021 | -5.95 | 0.000 | -0.166 | -0.084 |
| diabetes | -0.004 | 0.017 | -0.26 | 0.792 | -0.038 | 0.029 |
| heart | 0.016 | 0.019 | 0.84 | 0.400 | -0.021 | 0.053 |
| highbp | 0.002 | 0.013 | 0.16 | 0.876 | -0.023 | 0.027 |
| arthritis | -0.118 | 0.013 | -8.78 | 0.000 | -0.144 | -0.091 |
| overestweight | -0.142 | 0.051 | -2.81 | 0.005 | -0.242 | -0.043 |
| underestweight | -0.031 | 0.050 | -0.61 | 0.540 | -0.130 | 0.068 |
| overestuw | 0.158 | 0.079 | 2.00 | 0.046 | 0.003 | 0.314 |
| overestow | 0.038 | 0.090 | 0.42 | 0.676 | -0.139 | 0.214 |
| underestow | 0.096 | 0.055 | 1.76 | 0.079 | -0.011 | 0.203 |
| underestob | 0.087 | 0.056 | 1.57 | 0.117 | -0.022 | 0.197 |
| gintuw | -0.007 | 0.062 | -0.11 | 0.915 | -0.129 | 0.115 |
| gintow | 0.005 | 0.024 | 0.22 | 0.824 | -0.042 | 0.053 |
| gintob | -0.025 | 0.028 | -0.90 | 0.367 | -0.080 | 0.030 |
| gintoveruw | -0.040 | 0.095 | -0.43 | 0.669 | -0.226 | 0.145 |
| gintoverow | -0.012 | 0.107 | -0.11 | 0.914 | -0.221 | 0.198 |
| gintunderow | -0.206 | 0.080 | -2.57 | 0.010 | -0.363 | -0.049 |
| gintunderob | -0.064 | 0.078 | -0.82 | 0.413 | -0.217 | 0.089 |
| gintoverest | 0.035 | 0.059 | 0.59 | 0.555 | -0.081 | 0.150 |
| gintunderest | 0.113 | 0.071 | 1.60 | 0.110 | -0.026 | 0.251 |
| _cons | 0.765 | 0.025 | 30.05 | 0.000 | 0.715 | 0.815 |
| Number observations=1905 | |  |  |  |  |  |
| F(37,1868) = 22.57 | |  |  |  |  |  |
| Prob>F=0.0000 |  |  |  |  |  |  |
| R-squared=0.2826 | |  |  |  |  |  |
